# Microbiome Imbalance and Pediatric Type 1 Diabetes Mellitus: An Updated Systematic Review of Gut Dysbiosis Evidence

**DOI:** 10.7759/cureus.89279

**Published:** 2025-08-03

**Authors:** Francis Asante Baadu, Muhammad Ahsan, Bacha Hussain, Shandana Hussain, Hafsa Malik, Syeda Sarah Naqvi, Zain Mustafa, Maheen Zahid, Rana Taseer Ahmad

**Affiliations:** 1 Internal Medicine, Komfo Anokye Teaching Hospital, Kumasi, GHA; 2 Trauma and Orthopaedics, University Hospitals of Derby and Burton, Derby, GBR; 3 Medicine, Saidu Teaching Hospital, Swat, PAK; 4 Nephrology, Pakistan Institute of Medical Sciences, Islamabad, PAK; 5 Paediatrics, Federal Medical College, Islamabad, PAK; 6 Internal Medicine, Rawalpindi Medical University, Islamabad, PAK; 7 Paediatrics, Bakhtawar Amin Medical and Dental College, Multan, PAK; 8 Paediatrics, King Edward Medical University, Lahore, PAK; 9 Paediatrics, Mukhtar A Sheikh Hospital, Multan, PAK

**Keywords:** dysbiosis, gut microbiota, intestinal permeability, pediatric population, type 1 diabetes mellitus

## Abstract

Emerging evidence suggests that alterations in the gut microbiota may play a significant role in the development of type 1 diabetes mellitus (T1DM), particularly during childhood, when the immune and metabolic systems are still maturing. This systematic review aims to synthesize recent findings on the composition, diversity, and functional characteristics of gut microbiota in children with T1DM. A comprehensive literature search was conducted on PubMed, Cochrane Library, and Google Scholar for studies published between January 2019 and July 2025. Eligible studies included observational studies examining the gut microbiota in children with T1DM using validated sequencing methods. Six studies met the inclusion criteria and were analyzed for microbial composition, diversity, and associated immune and metabolic alterations. Most studies reported reduced microbial diversity and depletion of short-chain fatty acid (SCFA)-producing bacteria, such as *Faecalibacterium* and *Roseburia*, in T1DM children. An increased abundance of pro-inflammatory genera, including *Bacteroides*, *Blautia*, and *Dorea*, was frequently observed. Several studies have also identified elevated levels of gut permeability markers, such as zonulin and lipopolysaccharide (LPS), suggesting compromised intestinal barrier function. Notably, while some studies reported decreased abundance of *Parasutterella* in T1DM, one study observed its increased abundance, indicating regional or methodological variability. Gut dysbiosis, characterized by reduced diversity, loss of beneficial microbes, and increased intestinal permeability, is consistently associated with pediatric T1DM. However, the heterogeneity of specific taxa highlights the need for standardized longitudinal research. Understanding gut microbial alterations may provide novel opportunities for early intervention and disease modulation in at-risk children.

## Introduction and background

Type 1 diabetes mellitus (T1DM) is an autoimmune condition marked by the inadequate production of insulin due to the T-cell-driven destruction of pancreatic beta cells that secrete insulin [1]. Among pediatric populations, T1DM is one of the most prevalent autoimmune conditions. The incidence of T1DM peaks between the ages of 10 and 14 years, after which it gradually declines [2]. According to estimates from the International Diabetes Federation, over 1.8 million individuals aged < 20 years had T1DM in 2024 [3]. However, the incidence varies by country and region within a single nation. In many nations, the incidence of T1DM has increased over the past 50-60 years, increasing by approximately 3% annually [4,5]. With a notable rise in the 0-4 year age group, the disease is manifesting at a significantly earlier age in many nations [6], suggesting a lowered threshold for the development of T1DM.

Microbiota are groups of microorganisms that coexist harmoniously with their human or animal hosts [7]. The gastrointestinal tract, respiratory and urogenital systems, and skin contain symbiotic microbial cells that make up the human microbiota. Among these, the gastrointestinal tract harbors the densest microbial population, forming a complex community of approximately 4 × 10¹³ cells [8]. Although the gut microbiota includes a variety of microorganisms, bacteria have been the most extensively studied. One important technique for identifying and categorizing these bacterial communities is 16S ribosomal RNA (rRNA) sequencing [9,10]. Moreover, strain-specific and functional variations in the microbiome can be accurately identified using novel metagenomic techniques [11]. The majority of gut bacterial populations, or "core microbiota," are shared by healthy individuals. Only four phyla contain the majority of the thousands of bacterial species that have been isolated from the human gut: 90% of them are *Bacteroidetes *and *Firmicutes*, while *Actinobacteria *and *Proteobacteria *are less common [12]. However, evidence demonstrating a great deal of microbial variety over time and among human populations has called into question the idea of the "core microbiota" [13].

There is evidence that the pancreas and gastrointestinal tract are immunologically linked, which supports the significance of the gut immune system in the pathophysiology of T1DM [14]. Microbes, especially those found in the gut, may play a significant role in the pathophysiology of T1DM [15]. In T1DM patients, the *Firmicutes*/*Bacteroides* ratio is lower than that in healthy controls. Murri et al. [16] discovered that children with diabetes had much higher levels of *Clostridium*, *Bacteroides*, and *Veillonella* and significantly lower levels of *Lactobacillus*, *Bifidobacterium*, *Blautia coccoides*/*Eubacterium rectale* group, and *Prevotella*.

The interrelationship between gut permeability, the immune system, and intestinal microbiota is complex and remains an area of ongoing research [17]. The gut barrier, which regulates gut permeability, comprises several components, including gut bacteria, mucus, enterocytes, tight junction (TJ) proteins, and both innate and adaptive immune cells that constitute the gut-associated lymphoid tissue [15]. Increased intestinal permeability, sometimes known as leaky gut, is another hypothesized T1DM pathophysiology linked to microbial dysbiosis. It may function independently or in tandem with immune dysregulation [18]. Increased intestinal permeability due to TJ protein changes allows external agents (e.g., bacteria and bacterial and food products) to enter the lamina propria. When these bacteria and chemicals accumulate, they can trigger inflammatory pathways that result in intestinal inflammation. T1DM may be caused by enlarged and activated T-cells in the gut-associated lymphoid tissue traveling to the pancreas through the mesenteric and pancreatic lymph nodes [19].

While numerous studies suggest a potential link between gut dysbiosis and the onset of T1DM, current evidence remains largely associative, and causality has not been conclusively established. This systematic review aimed to compile recent findings on the connection between changes in gut microbiota and T1DM in children. The research explored the differences in gut microbiome composition and diversity between children with T1DM and those without the condition, while also assessing related functional and immune system changes, such as shifts in short-chain fatty acid (SCFA) production, intestinal permeability, and inflammatory markers. Additionally, this review seeks to identify consistent microbial patterns and variations reported across different geographic and demographic contexts. By integrating findings from observational studies that utilize validated microbiome sequencing methods, this review aims to clarify the potential role of gut dysbiosis in the pathogenesis of childhood T1DM and contribute to the foundation for future research and microbiota-based interventions.

## Review

Materials and methods

This review was conducted in accordance with the Preferred Reporting Items for Systematic Reviews and Meta-Analyses (PRISMA) 2020 guidelines. An extensive literature search was conducted using three major databases, namely, PubMed, Google Scholar, and the Cochrane Library, to identify studies examining the link between gut dysbiosis and T1DM in children. The search was restricted to articles published from January 2019 to June 2025 to reflect the most recent advancements in the field. Search terms combined both MeSH terms and free-text keywords. These included variations of the following terms: “gut microbiota”, “intestinal flora”, “microbiome”, “gut dysbiosis”, “type 1 diabetes mellitus”, “T1DM”, “children”, “pediatrics”, “infants”, and “adolescents”. Boolean operators "AND" and "OR" were applied to build relevant combinations and improve search precision. In addition, reference lists of selected studies and previously published reviews were hand-searched to locate any eligible studies that might not have appeared in the initial database results.

Inclusion and Exclusion Criteria

Studies were eligible for inclusion if they satisfied all of the following conditions: (1) they were original research articles employing observational designs, such as cohort, case-control, or cross-sectional studies; (2) they involved participants aged 0-18 years diagnosed with T1DM; and (3) they investigated gut microbiota composition or diversity using reliable analytical techniques, such as 16S rRNA sequencing or metagenomic analysis. Only studies conducted on human subjects and published in the English language were considered. The exclusion criteria were as follows: (1) studies involving adult populations; (2) reviews, editorials, commentaries, letters, and conference abstracts without full data; (3) animal or in vitro studies; and (4) studies not involving direct analysis of the gut microbiota or those lacking relevance to T1DM (Table 1).

**Table 1:** Inclusion and exclusion criteria T1DM: type 1 diabetes mellitus

Category	Inclusion criteria	Exclusion criteria
Population	Children and adolescents aged 0–18 years	Studies involving adult participants (>18 years)
Condition	Diagnosed cases of T1DM	Studies not involving T1DM or focusing on other metabolic/endocrine conditions
Microbiota assessment	Analysis of gut microbiota composition or diversity	Studies not directly assessing gut microbiota (e.g., immune markers only, metabolomics without microbiome data)
Methodology	Studies employing validated microbiome analysis methods (e.g., 16S rRNA gene sequencing, shotgun metagenomics)	Studies lacking validated or direct microbiome analysis methods
Study type	Observational studies (case-control, cohort, cross-sectional)	Reviews, editorials, commentaries, letters, conference abstracts, and animal or in vitro studies
Language	Full-text articles available in English	Articles not available in English
Publication date	Published from 2019 onward	Studies published before 2019

Study Selection and Screening

**Figure 1:**
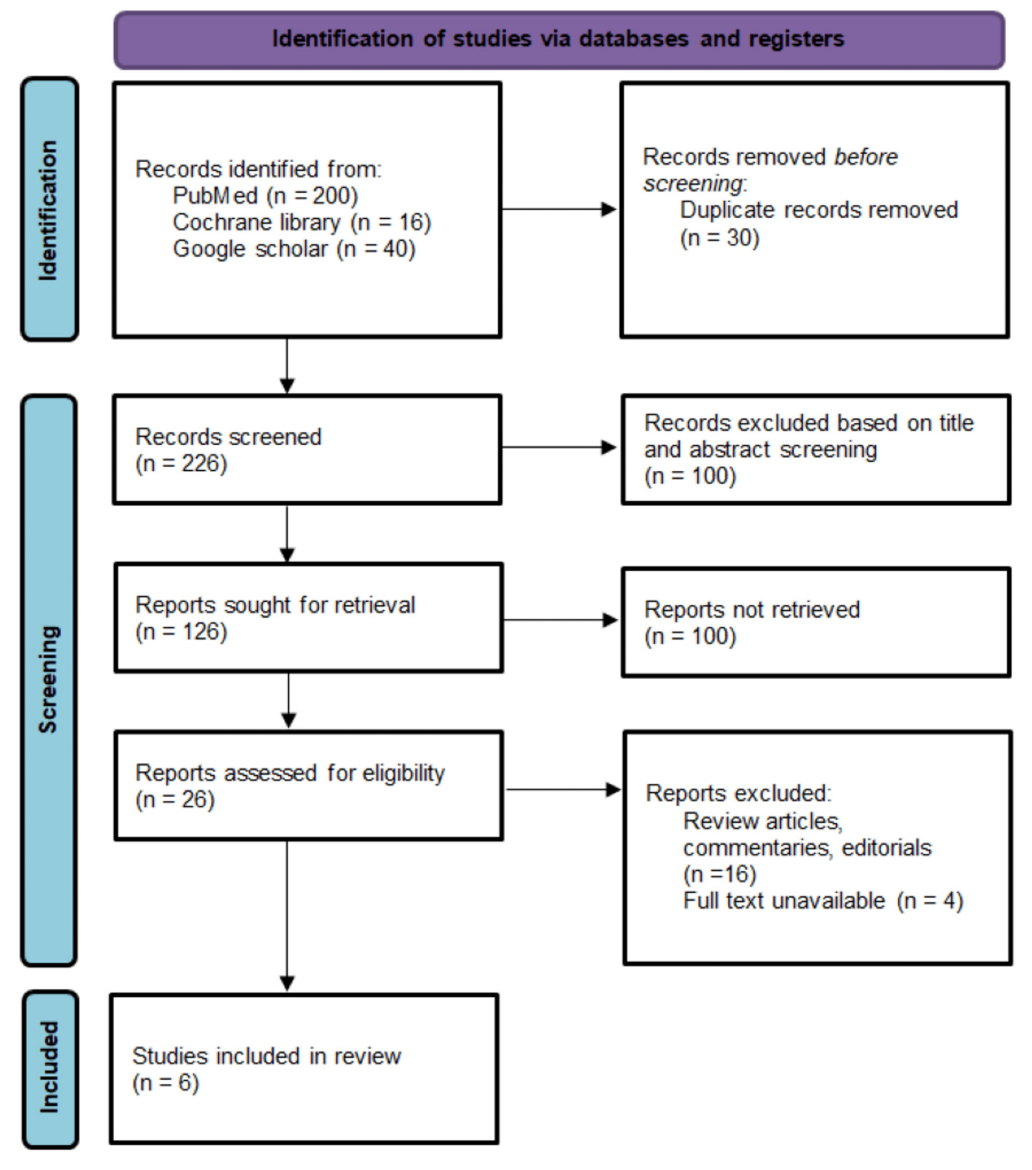
PRISMA flow diagram 2020 PRISMA: Preferred Reporting Items for Systematic Reviews and Meta-Analyses

All search results were imported into Zotero for organization, and duplicate entries were systematically removed. The screening process was carried out independently by two reviewers, who examined titles and abstracts to identify potentially relevant studies. For records that appeared eligible, full-text versions were obtained and evaluated using the established inclusion and exclusion criteria. Articles for which full-text access could not be obtained after multiple retrieval attempts, including institutional and open-source platforms, were excluded from final analysis. In instances where there was disagreement between reviewers, the issue was resolved through discussion or, when necessary, with input from a third reviewer. The PRISMA 2020 flow diagram illustrates the study selection process (Figure 1).

Data Extraction and Quality Assessment

A predesigned data extraction sheet was used to systematically collect essential information from each study. Extracted variables included: author names, publication year, and country of origin, study type, participant characteristics (age group, sample size), microbiome analysis method, and key microbiological or clinical outcomes associated with T1DM.

The methodological quality and risk of bias for each included study were assessed using the National Institute of Health (NIH) Quality Assessment Tool for Observational Cohort and Cross-Sectional Studies. This evaluation was conducted independently by two reviewers. Any discrepancies in scoring were resolved through mutual agreement after discussion.

Data Synthesis

Significant heterogeneity was observed across the included studies in terms of design, microbiota profiling techniques, population characteristics, and reported outcomes. As a result, quantitative pooling of data was not possible, and a meta-analysis was not conducted. Instead, a qualitative (narrative) synthesis was performed, highlighting consistent trends, microbial shifts, and functional implications of gut dysbiosis in pediatric T1DM.

Results

Out of the total 126 full-text articles identified for review, 100 could not be retrieved due to limited accessibility or incomplete availability. Of the remaining 26 articles screened in full, 21 were excluded - 16 did not fulfill the eligibility requirements, while five were inaccessible in full-text format. Following this rigorous screening process, six studies published between 2019 and 2025 were deemed suitable for inclusion in the final analysis. Details of these studies are summarized in Table 2.

**Table 2:** Summary of the reported cases of gut dysbiosis in children with T1DM (2019-2025) ↑: Increase in specific group; ↓: Decrease in specific group T1DM: type 1 diabetes mellitus

Author	Year	Design	Population Characteristics	Sample Size	Age Range	Microbiome Assessment Method	Key Microbial Findings	Immune Markers Measured	Major Conclusions
Harbison et al. [20]	2019	Prospective cohort	Children with islet autoimmunity (T1DM risk); progressors vs non-progressors	88	5–12 years	16S rRNA sequencing + permeability assays	↓ diversity, ↓ SCFA producers; ↑ permeability	Zonulin, Lipo-polysaccharide (LPS), SCFAs	Dysbiosis & leaky gut precede T1DM
Biassoni et al. [21]	2020	Cross-sectional	T1DM children vs. healthy	56	4-7 years	16S rRNA sequencing	↑ Bacteroides, ↓ Parasutterella	Autoantibodies	T1DM children showed altered genus-level abundances
Liu et al. [2]	2021	Cross-sectional	T1DM children vs. healthy	98	6–14 years	16S rRNA sequencing	↑ Eubacterium, ↓ Bacteroides	Not measured	Dysbiosis precede T1DM
Yuan et al. [22]	2022	Prospective Cohort	T1DM children vs. healthy	64	4–10 years	16S rRNA sequencing	↓ diversity, SCFA genes, ↓ Firmicutes, ↑ Bacteroidetes and Proteobacteria	interleukin-1 beta (IL-1β)/ LPS-binding protein (LBP)	Dysbiosis & functional loss
Tamahane et al. [23]	2023	Cross-sectional	T1DM children vs. healthy	129	9-15 years	16S rRNA sequencing	↑ Cyanobacteria, Parasutterella, ↓Acinetobacter as compared to controls	Not measured	T1DM children showed altered genus-level abundances
Kennedy et al. [24]	2025	Cross-sectional	T1DM children vs. healthy	100	6 months - 18 years	Shotgun metagenomic sequencing	↑ Bacteroides, ↓ Firmicutes	Serum zonulin, C-peptide levels	Dysbiosis precede T1DM

Study Characteristics

This review included six studies published between 2019 and 2023, comprising three cross-sectional studies and three cohort studies. The studies investigated pediatric populations with sample sizes ranging from 56 to 129 participants and age ranges from six months to 18 years old. All but one study used 16S rRNA gene sequencing to analyze the gut microbiota, with Harbison et al. [20] also assessing gut permeability using biomarker assays. Immune and inflammatory markers, such as zonulin, lipopolysaccharide (LPS), SCFAs, IL-1β, and LBP binding protein (LBP), were measured in three studies.

Gut Microbiota Composition and Diversity

Out of the six studies included in the review, four reported a notable decline in gut microbial diversity among children diagnosed with T1DM in comparison to healthy peers. These studies consistently demonstrated signs of microbial imbalance, primarily marked by a reduction in beneficial, SCFA-producing bacteria such as *Firmicutes* and *Faecalibacterium*. In contrast, there was a relative increase in pro-inflammatory microbial groups, particularly *Bacteroides*. Interestingly, one study [2] documented an elevated presence of *Eubacterium* alongside a reduction in *Bacteroides* within the T1DM group, while another [24] reported reduced *Firmicutes* and increased *Bacteroides*, consistent with dysbiotic signatures observed in the literature. However, Tamahane et al. [23] reported a higher abundance of *Parasutterella* in T1DM children, contradicting the findings of Biassoni et al. [21], suggesting potential geographic, dietary, or methodological differences.

Functional and Immunological Alterations

Three studies investigated the functional capacity of the gut microbiome and measured markers of gut permeability and immune activation. Harbison et al. [20] and Yuan et al. [22] both reported elevated levels of zonulin and LPS, indicative of increased intestinal permeability ("leaky gut"), in children with T1DM or islet autoimmunity. Functional metagenomic analysis by Kennedy et al. [24] revealed diminished SCFA biosynthesis pathways and a correlation with reduced serum C-peptide levels, suggesting the impact of microbial imbalance on β-cell function.

Discussion

This systematic review offers insights into the role of gut dysbiosis in the pathogenesis of T1DM in children. Our analysis of six studies highlighted important trends in the gut microbial composition, functional alterations, and immune interactions in pediatric T1DM. While our results partially align with global findings, they also reveal important divergences, potentially attributable to regional, dietary, or genetic factors.

A key finding of our review is that four of the six included studies reported a significant reduction in gut microbial diversity in children with T1DM compared to healthy controls. Similarly, de Goffau et al. [14] reported decreased abundance of *Bifidobacterium* species and SCFA-producing bacteria in children with β-cell autoimmunity, suggesting a disruption in microbiota beneficial for gut and immune homeostasis. Another study by de Goffau et al. [25] observed distinct microbial profiles in T1DM children, characterized by increased *Bacteroidetes *and reduced *Firmicutes *- a signature consistent with dysbiosis.

Notably, the study by Tamahane et al. [23] found no significant variation in alpha or beta diversity between children with T1DM and healthy controls. However, they did identify a significantly increased abundance of *Parasutterella* in the T1DM group (p < 0.05). *Parasutterella* has been linked to bile acid metabolism and host inflammation, though its role in T1DM remains unclear. This finding stands in contrast to several other studies, including Biassoni et al. [21] and Diviccaro et al. [26], both of which reported significantly reduced abundance of *Parasutterella* in T1DM patients compared to healthy controls. In those studies, *Parasutterella* - along with *Lactobacillus* and *Turicibacter* - was depleted in T1DM, suggesting a possible protective or homeostatic role in maintaining gut barrier integrity and immune balance.

Our review highlighted that three of the six included studies demonstrated increased intestinal permeability and altered microbial metabolic functions in children with T1DM. This is strongly supported by Harbison et al. [20] and Yuan et al. [22], who found elevated zonulin and LPS levels in diabetic children-markers of a "leaky gut" that may precede autoimmune responses. Kennedy et al. [24] further showed reduced SCFA biosynthesis and linked these changes to lower serum C-peptide levels, suggesting impaired β-cell function due to microbial metabolic dysfunction. These findings are in agreement with studies such as Alkanani et al. [27], who reported a distinct pro-inflammatory microbial signature in new-onset T1DM cases, including increased *Bacteroides* and reduced *Prevotella*, suggesting a shift toward carbohydrate-fermenting bacteria that may influence glucose metabolism and immune activation.

Evidence of disrupted gut barrier function is also well-established in the literature. Vatanen et al. [28], using metagenomic analysis in genetically predisposed children, showed that microbial communities in children who developed islet autoimmunity harbored fewer genes associated with SCFA production and exhibited pro-inflammatory functional signatures. Similarly, Leiva-Gea et al. [29] found elevated serum zonulin and endotoxin levels in children with T1DM, indicating compromised intestinal barrier integrity and microbial translocation. These alterations have been linked to immune priming and the acceleration of autoimmune responses in susceptible individuals. Consistently, de Goffau et al. [14] found that children with multiple islet autoantibodies had a lower abundance of SCFA-producing species such as *Faecalibacterium* and *Bifidobacterium*, and increased *Bacteroides*, supporting a functional shift from anti-inflammatory to pro-inflammatory metabolic output.

Furthermore, longitudinal studies strengthen the causal interpretation of microbiome changes preceding clinical onset. Kostic et al. [30], in a study tracking infants at genetic risk for T1DM, found that microbial diversity declined significantly prior to seroconversion, accompanied by a reduction in beneficial taxa such as *Roseburia* and *Bacteroides dorei*. Overall, findings from these external studies strongly support the idea that gut microbial imbalances - particularly involving pro-inflammatory taxa and functional disruptions in SCFA metabolism - are closely associated with T1DM development [31]. The convergence of evidence on increased gut permeability and reduced immunomodulatory microbial activity underscores the likely contributory role of the gut microbiota in the pathophysiology of pediatric T1DM.

Differences between our findings and those of international studies may stem from several factors. First, geographic variation in diet, hygiene, antibiotic exposure, and environmental microbiota can influence gut microbial composition [13]. Second, genetic and immunological differences among populations may modulate host-microbe interactions. Third, methodological differences, such as sequencing platforms and taxonomic classification algorithms, may affect the resolution and comparability of microbiota data. However, there are several limitations to this study. A notable limitation of this review is the unavailability of full-text access for a number of potentially eligible studies, which may have limited the comprehensiveness of the evidence base. Despite this, the included studies provided relevant and consistent findings that contribute meaningfully to the research question. Given the observational nature of the included studies, the findings should be interpreted as associations rather than definitive causal relationships. This inherent limitation restricts the ability to draw cause-and-effect conclusions.

The consistent involvement of SCFA-producing and pro-inflammatory taxa across multiple studies supports the potential for microbiota-targeted interventions in T1DM. Prebiotic or probiotic supplementation, dietary modulation, and even fecal microbiota transplantation have been explored as strategies to restore microbial balance. Future research should incorporate longitudinal, multi-omic approaches - including metabolomics, transcriptomics, and host immunophenotyping - to deepen our understanding of the gut-pancreas-immune axis. Additionally, studies involving underrepresented populations such as ours are crucial to building a globally inclusive understanding of T1DM pathogenesis.

## Conclusions

In summary, this review demonstrates a consistent association between gut dysbiosis and T1DM in children, characterized by reduced microbial diversity, altered abundance of SCFA-producing and pro-inflammatory taxa, and signs of increased intestinal permeability. While most studies support a pathogenic role of gut microbiota in T1DM, some conflicting findings - particularly regarding genera such as *Parasutterella* - highlight the influence of regional and methodological differences. Further longitudinal and functional studies are needed to clarify causality and inform microbiota-targeted strategies for early intervention and prevention in at-risk pediatric populations.
